# What do you mean “functional” in ecology? Patterns versus processes

**DOI:** 10.1002/ece3.6781

**Published:** 2020-10-15

**Authors:** Florence Volaire, Sean M. Gleason, Sylvain Delzon

**Affiliations:** ^1^ CEFE, Univ Montpellier, CNRS, INRAE, EPHE, IRD, Univ Paul Valéry Montpellier 3 Montpellier France; ^2^ USDA ARS, Water Management and Systems Research Unit Fort Collins CO USA; ^3^ Univ. Bordeaux, INRAE, BIOGECO Univ. Bordeaux Pessac France

**Keywords:** functional trait, genetic variability, life form, ontogeny, pattern, process

## Abstract

Use of the term “functional" trait has increased exponentially in ecology. Although accounting for numerous ecological questions, this concept raises several issues. We propose that the term “functional” could be misleading because (1) no rigorous criteria exist to identify “functional” traits and (2) it suggests that only some traits (“functional” ones) can inform our understanding of species functioning, whatever the scale or discipline. Hence, the concept of "functional" trait in ecology is starting to be challenged and it remains unclear why some traits should be considered functional, whereas other traits should not.

We argue that the most used “functional” traits are meaningful because they reflect important differences between populations or species, based on synchronic comparisons, that is, irrespective of time (hereafter “pattern” traits). Hence, they are useful for identifying trade‐offs and strategies across large numbers of observations, usually at rather coarse scales, and are most often used in analyses of “big data.” However, given that many ecological processes occur across short time scales and narrow gradients of climate and resource availability, the efficacy of these traits to inform us about these ecological processes appears questionable. We show that trait measurements that take time explicitly into account (hereafter “process” traits) differ from pattern traits because they quantify the flows of material and energy within a given environment across a defined period of time. Although pattern traits and process traits are both functional, it is important to understand the differences between the approaches. Moreover, better accounting of ontogeny, life form, plasticity, and genetic variability is required to enhance the convergence between pattern and process approaches. This revised framework allows more explicit connections between trait ecology and other biological sciences. It should enhance the study of processes at all scales in order to investigate efficiently the adaptive responses of biological organisms to climate change.

## Graphical abstract

Revising the concept of "functional" trait: In ecology, pattern approaches based on "functional" traits that do not explicitly take time into account, differ from process approaches which measure response curves at all scales over time. Using the term “functional” for traits meaningful for both pattern and process approaches, and better accounting of ontogeny, life form, plasticity and genetic variability should enhance the convergence across approaches within biological sciences.

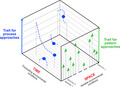

Understanding patterns in terms of the processes that produce them is the essence of science (Levin, 1992).

## 1 INTRODUCTION: REVISING THE CONCEPT OF 'FUNCTIONAL TRAIT'

In ecology, the concept of the "functional" trait underpins trait based ecology. "Functional" traits are “any features measurable at the individual level, without reference to the environment or any other level of organization, and which impact fitness indirectly via their effects on growth, reproduction and survival” (Violle et al., 2007). For the last two decades, the number of publications including the term "functional" trait has increased exponentially, corresponding to more than 20% of the publications in plant ecology over the last 4 years, compared to only 1% in the 2000s (Web of Science). Yet, a large majority of studies involving the measurement of traits in organism, community, or ecosystem ecology only refer to the term trait and not to the term “functional" trait. We argue that distinguishing between different meanings, here of traits and “functional" traits, is of much more than academic or semantic interest (Jax, 2005). From the gene to the whole organism, scientists measure—and have always measured—a large range of traits (called for instance “variables,” “parameters,” or “characteristics”) to understand biological functioning. Beyond the field of ecology, these traits are not termed “functional” although most traits commonly quantified from alleles to whole organisms are “functional,” that is, provide information on plant functioning. Therefore, using the term “functional" trait only in the field of ecology creates a semantic obstacle to multi‐scale approaches and collaboration between disciplines within the plant sciences (ecophysiology, agronomy, plant genetics, etc.) and implicitly suggests that “functional” traits are the only traits that can inform our understanding of plant functioning. Since, functional approaches encompass all biological sciences aiming to understand functions, process, and patterns from the organismic (genetics, physiology, and ecophysiology) to the ecosystem scale (ecology, ecosystems ecology, and evolutionary biology), we need concepts that are valid for all these disciplines. We propose to define a trait as *a variable measured on an organism at any scale, from gene to whole organism and which can be scaled up from individuals to genotype, population, species, or community* (Box 1).

Box 1 **Revised terminology**

- **Trait** = a variable measured on an organism at any scale, from gene to whole organism and which can be scaled up from individuals to genotype, population, species, or community.
- **Functional trait** = a trait arising from or influencing an organism’s fecundity, growth, development, or survival, that is, demographic fitness.
- **Trait for pattern approach (pattern trait)** = a trait measured in standardized (comparable and therefore generally optimum) conditions, irrespective of time (e.g., traits of the leaf economics spectrum).
- **Trait for process approach (process trait)** = a trait measured under environmental conditions fluctuating in time, which characterizes processes, that is, flows of material and energy in a given environment during a defined period of time (e.g., traits accounting for seasonal adaptation, responses to biotic or abiotic stress or perturbation).
- **Function** = the “movement or storage of energy or material” from a cellular to a global level (Bellwood et al. 2019)

Despite its foundational importance, the practical difficulty of documenting trait–fitness relationships suggests that a trait's relationship to fitness cannot be used as a practical criterion for choosing “functional” traits (Shipley et al., 2016). Hence, the distinction between trait and "functional" trait appears not to be based on objective criteria because most measurable traits of plants and animals are likely to play a role in organism functioning. Therefore, among all traits, which should we consider “functional,” and therefore ecologically important? Are all traits "functional"? Are only some of them "functional"? Or, are some traits more "functional" than others? There appears to be no rubric to assist us in answering these questions, suggesting that either the term “functional” has no real meaning, at least not a rigorous or intuitive one, or else the concept of ecological functionality itself requires revision.

Here, we re‐evaluate how we define “functional" trait, with the purpose to encourage more explicit connection within ecology and between trait ecology and other biological sciences. We mainly illustrate our discussion with the example of plant science since the term “functional" trait was initially coined in plant ecology. However, we suggest that our views are broadly relevant to any functional approaches whatever the biological model, for example (Menezes, Baird, & Soares, 2010; Moretti et al., 2017; Pigot et al., 2016).

### 1.1 What do you mean 'functional'?

In plant science, variation of “functional” traits within and among species is assumed to reflect ecological information, such as life history strategy (Adler et al., 2014), community assembly (Laughlin, 2014; Lavorel & Garnier, 2002; Westoby & Wright, 2006), response of communities to disturbances (Mouillot, Graham, Villeger, Mason, & Bellwood, 2013), species evolution and adaptation (Larter et al., 2017), species distribution and niche conservatism (Wiens et al., 2010), and services provided by agro‐ecosystems (Diaz et al., 2013; Wood et al., 2015). Modern trait ecology represents a notable advancement from the first qualitative and semi‐quantitative efforts to understand plant form and function (Gleason, 1926; Grime, 1979). The primary advancement being the placement of measured traits directly onto quantitative axes, representing meaningful trait‐space dimensions (Westoby, 1998). Subsequent efforts from this time have largely extended this initial idea to a wider range of species and habitats. Many studies have successfully applied trait ecology to improve our understanding of plant–plant and plant–environment relationships at the global scale (Choat et al., 2012; Wright et al., 2004) and plant functional diversity across the vascular plant phylogeny (Cornwell, Bhaskar, Sack, Cordell, & Lunch, 2007). Central to these efforts is the identification of traits that can be easily measured, such that hundreds/thousands of taxa can be represented, but which are also informative of important ecological processes (Cornelissen et al., 2003). An example of this is the TRY database (Kattge et al., 2011), which represents a global effort to gather together easily measured yet informative traits in one accessible place. Based on the comparative study of a large number of species (Diaz et al., 2016), this approach aims to analyze structural relationships among traits, that is, bold and regular patterns which are central in ecology (Lawton, 1996). However, these broad scale patterns of plant functioning reflect the underlying trade‐offs that have arisen from the struggle for finite resources, and are therefore likely to change as species, resource availability, and climate change with time (Rosado, Dias, & de Mattos, 2013). Understanding and predicting ecological processes from species’ traits is a “Holy Grail” that is starting to be challenged (Funk et al., 2017; Paine et al., 2015; Yang, Cao, & Swenson, 2018). They postulate that a few standardized key traits (“functional” traits), possibly measured at one time during a plant's life, can account for the most relevant ecological processes affecting the distribution and abundance of species appears questionable.

The limitations of "functional" traits in explaining important ecological processes are well known in the literature. For example, some of the most widely studied "functional" traits in plant ecology (specific leaf area, wood density, and seed mass) were found to be poor predictors of tree growth across the earth's terrestrial habitats (Paine et al., 2015). It is likely, however, that a more mechanistic understanding of climate‐species linkages would improve not only our ability to predict growth, but also other processes affecting community dynamics (Craine, Engelbrecht, Lusk, McDowell, & Poorter, 2012). It has also been suggested that our inability to measure the relevant traits underpinning population‐level demographics represents a significant challenge to trait‐based ecology and its ability to address the most meaningful ecological questions (Yang et al., 2018). This is not a subtle point of disagreement, but rather, indicates that our approach to understanding the linkages between traits and organism‐level performance requires revision (Kraft, Metz, Condit, & Chave, 2010). Within the same vein, a comprehensive review of the role of "functional" traits (Funk et al., 2017) concluded that the utility of trait‐based approaches in ecology would benefit from efforts that demonstrate how traits influence organismal, community, and ecosystem processes across vegetation types. Some authors have also recently stated that we should progress from “functional” to mechanistic traits (Brodribb, 2017) and that many important physiological traits have not yet been incorporated into the functional approach (Medeiros et al., 2019).

The term “function” in ecology has been defined according to the level of complexity of the considered system (Jax, 2005). In many instances, the term “function” refers to “state changes in time and is a synonym of *process”* (Calow, 1987; Jax, 2005). A function is more precisely defined as the “movement or storage of energy or material” from a cellular to a global level (Bellwood, Streit, Brandl, & Tebbett, 2019). However, given that ecological systems represent a complex system of interactions, our understanding of how they “function” requires an explicit understanding of both the *state* and the *trajectory* of the system (Jax, 2005). We propose then that *the term “function” should include both patterns and processes in ecology*.

**Figure 1:**
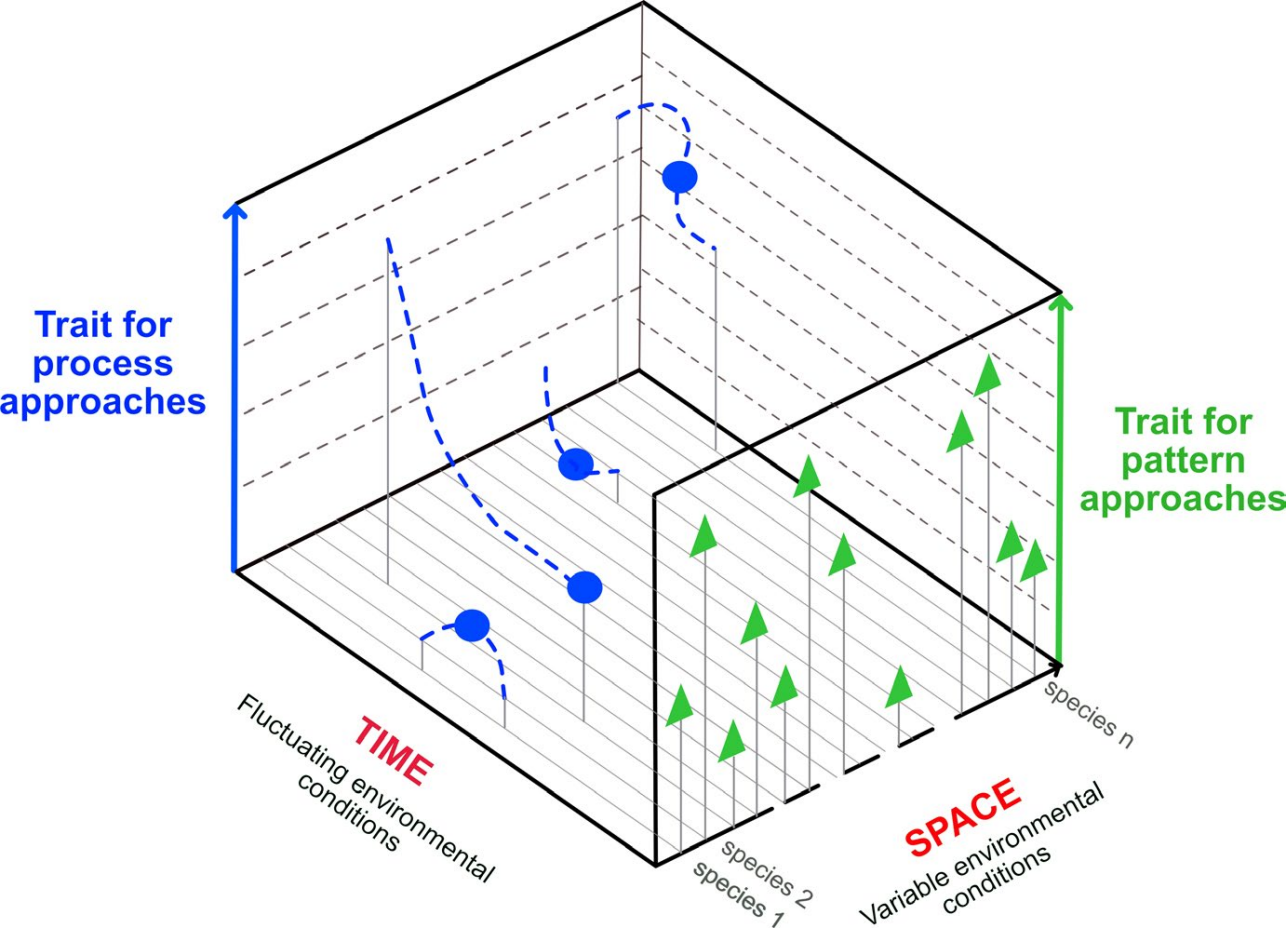
Schematic representation of traits measured across a number of individuals of different species (and/or genotypes, populations): Pattern traits (green triangles) accounting for variability of individuals functioning at a given time and across spatial gradients of environmental conditions (e.g., specific leaf area, plant maximum height), and different process traits (blue circles) measured under fluctuating environmental conditions and across a relevant period of time (e.g., plant growth rate, dynamics of leaf water potential, accumulation of solutes, and variation in photosynthetic activity). The representation of blue circles is arbitrary but shows a range of characteristic values (e.g., slope, inflexion point, minimum) of mathematical functions established between a biological response and time or variation in environment (blue dotted lines). Considering that many process traits are difficult to measure, we have included fewer blue circles than green triangles

In this context, it is noteworthy that regarding "functional" traits, “there is some lively debate surrounding definitions of terms,” and in particular whether, or not, rates measured over very short time scales versus rates measured over long time scales should be equally considered as "functional" traits (Walker, McCormack, Messier, Myers‐Smith, & Wullschleger, 2017). The TRY database for instance includes less than 3% of rate variables expressed *as a function of time* (65 in 2,100 traits), and most of them are not independent (e.g., same trait repeated per day, per month, and per year). Yet it remains unclear why some traits should be considered "functional", whereas other traits should not. We argue that a workable solution to this problem may be to recognize that *traits differences between species or populations may not accurately reflect temporal responses to fluctuating biotic and abiotic conditions*. Hence, we propose to distinguish between pattern approaches that do not explicitly take *time* into account, from process approaches, which measure response curves at any scale (from organ to community) over time (Figure 1). Consequently, we suggest *to revise the terminology and use the term “functional” for both pattern and process approaches*. Among all traits, we therefore propose to define a functional trait, as *a trait arising from or influencing an organism's fecundity, growth, development, or survival, that is, demographic fitness*. We note that this definition includes both pattern and process approaches, but differs from Violle et al. (2007) because it does not include *“*without reference to the environment or any other level of organization". Our proposed definition therefore does not seek to make traits more comparable between species by minimizing the effect of local environments (Cornelissen et al., 2003; Moretti et al., 2017), as this condition specifically refers to traits used for pattern approaches (See 1.2).

In science, the distinction between patterns and processes is by no means novel although it has been relatively little used in ecology (Levin, 1992). Whereas this distinction is important in other disciplines (physics, astrophysics, and social sciences), we argue that *the term “functional” trait, if referring mainly to traits for pattern approaches, blurs the heuristic distinction between patterns and processes in ecology*. In addition, as “functional” traits have been widely used, it is questioned whether the use of such “fashionable” traits is always justified (Rosado et al., 2013). We propose that recognizing the distinction between pattern and process approaches may help to clarify this current debate in functional ecology because it forces us to think more carefully about what processes have engendered a particular trait, and therefore, how it might be used appropriately in an ecological context.

### 1.2 Patterns versus processes

Plant “functional” traits, even termed "integrative" traits (Volaire, 2018) or “classic” traits (Maréchaux, Saint‐André, Bartlett, Sack, & Chave, 2020), have been defined and used successfully to identify key dimensions of variation (and therefore functioning), both within and across species. The most common traits used toward this purpose have been specific leaf area (SLA; fresh leaf area per unit dry leaf mass), leaf dry matter content (LDMC), leaf life span, leaf nitrogen and phosphorus concentration (or content), specific root length (SRL), maximum photosynthetic rate in optimum conditions, wood density and maximum plant height. These traits can be aggregated from individual to community levels as “community functional parameters” (Violle et al., 2007). Arguably, the most important trait variation axis that has been identified is the leaf economics spectrum (LES), which describes a continuum of plant strategies that span a range from slow return on N and C investment to a fast return on these investments (Wright et al., 2004). Since this time, LES‐like spectrums have been identified for roots (Comas & Eissenstat, 2004; Roumet et al., 2016), wood (Chave et al., 2009), and whole plants (Reich, 2014). These studies report covariation between traits, usually across diverse taxa and habitats, which identify regular patterns (Lawton, 1996) and are thus termed “universal” and “worldwide” (Wright et al., 2004). One advantage to this approach is that environment, life form, and other trait/environment characteristics can be plotted on the same axes to determine whether they are aligned with this covariation or are orthogonal to it (Wright et al., 2004). A disadvantage is that finer‐scale processes and temporal dynamics cannot be better understood. We describe these studies as pattern approaches because they are based on (mostly) one‐time trait measurements that underpin robust and meaningful trade‐offs (Lawton, 1999). These approaches identify structural, morphological, or physiological traits and strategies that have been shaped by evolutionary processes, and which account for meaningful proportions of variation in resource acquisition and/or conservation, and therefore help explain differences in fecundity, growth, and survival across resource gradients (Reich, 2014). “Functional” traits have been used to identify major axes of variation across species, perhaps most successfully via meta‐analyses and “big data” (Diaz et al., 2016). This approach can integrate a very large functional diversity if the data are sufficiently stratified (e.g., across habitats/phylogeny/physiology) (Walker et al., 2017). Functional diversity patterns can also help explain community assembly (Lebrija‐Trejos, Perez‐Garcia, Meave, Bongers, & Poorter, 2010; Spasojevic & Suding, 2012) when taking into account the relevant dimensionality of plant traits (Laughlin, 2014), which—we underline—does not include time. Based on Big Data Biology (Callebaut, 2012), this approach aims to understand the patterns, causes and consequences of biodiversity. In ecology, the traits that have been identified for these approaches give a synchronic “snapshot” of the observed variability at a given time. We can therefore propose to define traits *for pattern approaches as traits measured in standardized (comparable and therefore generally optimum) conditions, irrespective of time* (Box 1, Table 1). Conceptually, this approach resembles “structuralism” in anthropology, which aims to understand relevant structures, that is, the major dimensions and patterns underpinning diverse forms of social cultures irrespective of their history (Lévi‐Strauss, 1958).

On the other hand, the diachronic approach aims to understand how organisms acquire and use resources for metabolism, movement, growth, reproduction, and survival, as well as how ecosystems cycle, store or lose resources through biotic and abiotic processes. For practical reasons, this mechanistic and diachronic approach generally applies so far to a limited number of species, populations, genotypes, or communities. We note that the diachronic approach requires that traits are measured as a function of time, and therefore often requires repeated trait measurements across time. These traits are usually measured as the response to environmental factors that change in time (to calculate response curves). They represent characteristic values (e.g., slope, inflexion point, minimum, maximum, and thresholds) of mathematical functions established between a biological response and time or variation in environmental factors. We can define these traits for process approaches (Box 1, Table 1) as *traits measured under environmental conditions fluctuating in time, which characterize processes, that is, flows of material and energy in a given environment during a defined period of time (e*.*g*.*, seasonal adaptation, responses to biotic or abiotic stress or perturbation)*. The process approach is undoubtedly functional since it accounts for the change in materials or energy across *time*. If the traits of pattern approaches constitute the “warp” of plant diversity, as it has been proposed (Walker et al., 2017), the traits of process approaches can be regarded as the “weft” that equally contributes to the fabric of plant diversity. By analogy with the social sciences, the diachronic approach is similar to the study of history, or experimental social sciences, which seek to understand how the past influences the trajectory of current events.

Process traits are more difficult than pattern traits to be included into large comparative databases because environmental variation during the period of measurements should be taken into account to fully understand the meaning of the traits and to compare them across sites and experiments. Conversely, pattern traits that best account for the diversity of species and communities (e.g., economics spectrum traits) are mainly used in databases *irrespectively of the time and the state of the environment that occurred when they were measured*. They differ meaningfully from process traits that account for temporal variations in the flows of water, carbon, energy, and minerals (e.g., water use, transpiration rate, carbon assimilation, growth rate, mineral uptake rate, variation in organ temperature, dynamics of senescence, and phenological stage duration). This is because process traits can be measured across discrete time intervals, and therefore inform about processes and mechanisms that also operate across these time intervals (e.g., gene expression, post‐transcriptional regulation, and biochemical functioning).

These process traits are essential for parameterizing models that quantify fluxes of carbon, water and energy and species dynamics at different spatial and temporal scales (day, season, and year), generally during important developmental periods (growth, dormancy, and annual cycle) or during periods of environmental change (biotic or abiotic stress).

As such, we suggest that both pattern and process approaches contribute to our understanding of ecology and physiology and we therefore offer a few key points to facilitate the convergence and complementarity of these approaches.

**Table 1:** Comparison of Pattern versus Process approaches

	Pattern Approach	Process Approach
Dominant scope	Structure–networks–patterns	Mechanisms–processes
Main approach	Integrative Multivariate analysis—covariation between traits Many biotic and abiotic environmental factors	Mechanistic Response curves Limited number of biotic and abiotic environmental factors for controlled experiments but high “*in natura”*
Main methods	Synchronic Comparative study of individuals at similar ontogenic stage, often in optimum environmental conditions Snapshot of a large number of individuals at a given time	Diachronic Analysis of plant responses under fluctuating biotic and abiotic factors Study of dynamic processes as a function of time in limited numbers of individuals, populations, and communities
Dominant dimension	Space Spatial comparisons of “static” traits	Time Analysis across time of “dynamic” traits
Major traits	Traits encapsulating the main integrative functional strategies of individuals irrespective of time (and scaled up at populations, species, and communities levels)	Traits accounting for time variation in environmental conditions Characteristic values of response curves (e.g., minimum, maximum, slope, inflexion points, and threshold)
Dominant scale	Species to ecosystems	Genes to ecosystems
Data set	Large and required minimum number of species/populations/communities	Small number of—and even valid for a single—species/population/community
Major outputs	Identification of gradients, strategies, and trade‐offs of resource acquisition versus resource conservation	Quantification of main carbon, water, and mineral flows from organs to ecosystems
“Fabric of life” analogy	Functional warp of plant diversity	Functional weft of plant diversity
Analogy with social sciences	Structuralism	History, experimental social sciences

### 1.3 Plant traits and time

Phenotypically plastic traits, developmental trajectories, and gene expression, all change with environmental conditions and age (Stinchcombe & Kirkpatrick, 2012). However, pattern approaches in ecology involve the measurement of a few traits on relatively few standardized individuals (Pérez‐Harguindeguy et al., 2013), usually at organ level (for instance mature and healthy leaves exposed to full light) and often only once (for instance, at late successional stages for plant communities). We argue that these common practices raise a number of questions.

Firstly, plant response to different levels of resource shortage and stress is mainly mediated through whole‐plant processes that vary with time, for example, biomass allocation, growth reduction and modulation of organ development (Falster, Duursma, & FitzJohn, 2018). However, these “size related” traits are rarely considered because they are not easy to measure or assess. Yet, they may well capture much of an individual plant's response to stress. Traits measured on one organ cannot take into account ontogenic changes at the whole‐plant level (leaf/stem ratio, reproductive/vegetative ratio, relative importance of tillering, sprouting, etc.). For instance, life span, clonal growth, and resprouting across herbaceous species showed a stronger relationship with the environment than the major Leaf‐Height‐Seed FT axis (Herben, Klimesova, & Chytry, 2018). Similarly, leaf area index or whole‐plant leaf area is a more relevant trait than specific leaf area for understanding plant‐level and ecosystem‐level responses to herbivory and water supply (Brodribb & Hill, 2000; Gleason, Blackman, Cook, Laws, & Westoby, 2014). This applies to root systems as well because measuring traits on the most accessible roots (in the 20 cm top layer of the soil) may provide a biased assessment of root system functioning (Iversen et al., 2017). For instance, soil depth strongly influences fine root trait values across a range of grassland species (Fort et al., 2017). Consideration of roots and resource uptake at depth, as well as linking root form to function, are now recognized as keys to understanding whole‐plant functioning (Tumber‐Davila & Malhotra, 2020). Just as phylogenetic questions are often considered in plant trait studies, we suggest that ontogeny represents a similar opportunity to understand trait relationships since it is increasingly suggested that trait–trait or trait–environment correlations change with time and ontogeny (Charrier et al., 2018; Damian, Fornoni, Dominguez, & Boege, 2018). Crop physiologists, agronomists, and breeders take developmental processes into account when measuring process traits because they determine yield quantity and quality, as well as biotic interactions arising from resource competition.

Secondly, traits measured once in the life of a plant cannot wholly account for response to variation in resource availability. For example, considering the large range of plant strategies that have arisen to cope with drought (Volaire, 2018), even in the same community (Craine et al., 2013), it is doubtful that measuring a few integrative static traits can rightly account for this diversity (Yang et al., 2018). In most natural environments, resources fluctuate daily and seasonally, as do plant traits (Forner et al., 2018). Therefore, a problem that arises when so called “functional” traits are assumed to be “functional” whatever the ecological context and the research hypothesis, is that they tend to be regarded and used in process approaches as well. Most traits used for pattern approaches are not suitable to understand short‐term processes because they are generally either instantaneous measurements (e.g., maximum photosynthetic capacity), or else integrate plant response over an often uncertain period of time (e.g., carbon isotope discrimination). For instance, leaf dry matter content would be a poor choice to quantify the effects of a short‐term drought because it changes too slowly, whereas a trait such as tissue relative water content might represent a more meaningful response to drought (Saura‐Mas & Lloret, 2007). Many “classic” “functional” traits of pattern approaches such as SLA and LDMC are used to quantify drought response, rather than more responsive traits that are more proximally related to drought, such as the minimum operating water potential, level of xylem embolism, and the hydraulic safety margin (Anderegg et al., 2016). Considering that the most commonly measured “functional” traits are not suitable for predicting species’ responses to quickly fluctuating resources (e.g., water) (Brodribb, 2017; Griffin‐Nolan et al., 2018), the choice of traits should ideally be based on ecophysiological knowledge (Rosado et al., 2013). Pattern traits are most often substituted for more informative traits when they are easier and less expensive measure. However, we suggest that it is usually not suitable to simply measure pattern traits across shorter time intervals (such as the rate of variation of LMA, SLA, or maximum plant height) because these traits will rarely be meaningful across these intervals. Conversely, in process approaches, variation in traits such as photosynthetic rate, hydraulic conductance, tissue water content, water potential, or the concentration of metabolites is most meaningful when measured across the range of the resources encountered by the plant (e.g., soil water potential) and across a relevant time scale (Bouzid et al., 2019; De Long et al., 2019; Reich, Hobbie, Lee, & Pastore, 2018; Roy et al., 2016).

### 1.4 Plant traits and genetic variability

The use of continuous trait distributions within a plant community is a common way to implement the pattern approach, but this approach also has drawbacks. Firstly, moving from taxonomy and phyto‐ecology, which recognizes the importance of phenology (e.g., annuals, perennials, woody, and herbaceous) and within‐species variation, functional ecology often trades this information for a larger sample size, and thus loses key ecological dimensions that affect our interpretation of trait relationships (Raunkiaer, 1934). A common dimension‐reducing practice is to categorize taxa into plant functional groups. Such classification systems may be arbitrary, “traditional,” or be too broadly defined to provide insight into important levels of trait variation (Funk et al., 2017). In contrast, plant life form offers rigorous and meaningful species categories. Depending on the objective of the study, life form can be a crucial trait to consider when attempting, for instance, to link traits and ecosystem services, that is, when life forms or groups of species (e.g., annual plants, legumes, woody plants, and flowering plants) contribute disproportionately to ecosystem services (e.g., nitrogen fixation, productivity, or pollination). For example, a study examining the change in SLA or leaf nitrogen concentration in plants along an environmental gradient should also consider differences in life form (e.g., the proportion of annuals or N‐fixing species) to fully explain the ecological meaning of the results.

Secondly, when considering the relationships between plant traits and environment, traits measured on individuals of the same species are often merged in single datasets under the generic term “intra‐specific variability” (Jung, Violle, Mondy, Hoffmann, & Muller, 2010; Siefert et al., 2015; Violle et al., 2012). Consequently, variability between organs of a same plant, variability between different plants of a same population, or even variability between genetically different populations of the same species are equally ascribed to a similar “intra‐specific variability,” while variation between and within populations can be markedly different (Lamy et al., 2011). As a result, genetic variation and phenotypic plasticity, as well as their interactions, are not recognized nor identified, that is, this approach cannot properly account for plant–environment relationships (Kremer, Potts, & Delzon, 2014). Identifying and considering the different levels of plant trait variability (intraplant, within‐population, interpopulation, and interspecies), could enhance the detection of more robust ecological patterns, and thus better account for plant–environment interactions. This approach would be particularly useful when designing large databases that combine plant traits from a wide range of environments. Moreover, the overall quantification of the ratio between intra/interspecific variability from databases should take into account this issue. In particular, it would be sound to compare traits of species (interspecific variability) and traits of distinct populations (intraspecific variability) only when populations originate from the entire distribution range of the species. This would allow evaluation of a species’ capacity to exhibit different values of either pattern or process traits, for instance in the face of climate change, either via plasticity from within a given population, or via the migration of intrinsic variation from outside the population.

## 2 CONCLUSION AND PERSPECTIVES

The ability to make trait measurements across large numbers of species/populations and identify general patterns, trades‐off with the ability to obtaining a deeper understanding of physiological and ecological processes on fewer species/populations. We argue here that *traits suitable for pattern approaches should not be mistaken for traits suitable for process approaches*. However, the complementary between these approaches will likely increase as our ability to measure traits across time also increases and thus our application of the process approach (e.g., high throughput phenotyping and “omics” molecular ecology (Creer et al., 2016)). Process approaches at large spatial scales, across many species/populations, and at different times represent an important opportunity to better understand ecological processes *in natura* (Brodribb, 2017). Moreover, process approaches that explicitly take time into account and that also perform measurements across environmental conditions (e.g., higher atmospheric CO_2_, extreme temperatures, and combinations of factors) may be more relevant than pattern approaches for predicting the response of plants and plant communities to novel environments (Hanson & Walker, 2019; Korell, Auge, Chase, Harpole, & Knight, 2019; Song et al., 2019). While most approaches are correlative, standardized mechanistic approaches (Johnston et al., 2019) such as process‐based models (Journe, Barnagaud, Bernard, Crochet, & Morin, 2020) and manipulative physiological experiments (Lembrechts, 2020) are strongly advocated to inform environmental policy decisions in the face of climate change. Assessing the magnitude of phenotypic plasticity is crucial for predicting the responses and potential evolutionary resilience of organisms under climate change (Sultan, 2000, 2007). Many central questions in ecology and evolutionary biology indeed require characterizing phenotypes that change with time and environmental conditions (Vitasse, Bresson, Kremer, Michalet, & Delzon, 2010). To this end, suitable traits are inherently functions—defined here as process traits—and for instance, new “function valued” methods have been proposed to include a dynamic dimension to reaction norms in order to better account for the continuous change in traits of interest (Stinchcombe et al., 2012) and for studying how organisms can cope with environmental shifts (Nicotra et al., 2010).

Mathematical models, field manipulation experiments, and the search for large‐scale patterns are all valid approaches, and all have their strengths and weaknesses (Lawton, 1996). As recently proposed, we need to conduct extensive research to understand how much and why parameters vary over space and time, that is, what are both the patterns and the mechanisms underlying parameter variations (Luo & Schuur, 2020). Specifically, we show that *pattern and process approaches are orthogonal (space versus time) and therefore cast a complementary light on plant biology and ecology*. Hence, pattern and process traits need to be both evaluated in multiple environments to deliver robust inference on functions. However, their respective scopes and limitations should be recognized so that they can become truly complementary. Nevertheless, *we advocate that* *for a trait to provide relevant insight into the functioning of any species/population or integrated at community levels, researchers would benefit by considering carefully the choice of pattern* *versus* *process* *approaches, according to the scope of the research objectives*. To this end, we highlight a few outstanding questions (Box 2) as key perspectives to encourage more explicit connection within ecology, and between trait ecology and other biological sciences.

Box 2 **Perspectives—Main methodological issues**

- Depending on the research questions, can recommendations be made to specify the minimum number of populations, species, and range of environments necessary to ensure the optimum relevance of pattern approaches?
- Scaling up the approaches is needed to understand plant functioning: How much can pattern approaches at organ levels account for processes and plant functioning at the whole plant and ecosystem levels?
- Considering that plant traits vary across ontogenetic stages, across spatial and temporal scales, between organs and whole plants, and across individuals and populations, how can pattern and process studies be better designed to consider these sources of variation? 
- Considering that processes are being increasingly measured using high throughput phenotyping methods, potentially accounting for variation across large spatial and temporal scales and across many species/populations, might some process traits be candidates for pattern traits in the future?

Levin (1992) proposed that our theoretical understanding of ecosystem and community organization must include the quantification of patterns within and across systems. Functional ecology has undoubtedly met this target over the last two decades through the identification of broad scale trait patterns. Considering that the key to understanding ecological process, and thus, predicting process‐level change, lies in the elucidation of the mechanisms underlying observed patterns (Levin, 1992), we think it is now time for us to focus on the study of processes and process traits.

## CONFLICT OF INTEREST

None declared.

## AUTHOR CONTRIBUTION

**Florence Volaire:** Writing‐original draft (equal). **Sean M Gleason:** Writing‐original draft (equal). **Sylvain Delzon:** Writing‐original draft (equal).

## Data Availability

No data.
